# Cerebral Perfusion Pressure-Guided Therapy in Patients with Subarachnoid Haemorrhage—A Retrospective Analysis

**DOI:** 10.3390/life13071597

**Published:** 2023-07-21

**Authors:** Agata Gradys, Jakub Szrama, Zsolt Molnar, Przemysław Guzik, Krzysztof Kusza

**Affiliations:** 1 Department of Anaesthesiology, Intensive Therapy and Pain Management, Poznan University of Medical Sciences, 60-355 Poznan, Poland; agata.gradys@usk.poznan.pl (A.G.); k-kusza@wp.pl (K.K.); 2 Department of Anaesthesiology and Intensive Therapy, Semmelweis University, 1085 Budapest, Hungary; 3 Department of Cardiology, Intensive Therapy, Poznan University of Medical Sciences, 60-355 Poznan, Poland; pguzik@ptkardio.pl

**Keywords:** subarachnoid haemorrhage, cerebral perfusion pressure, intracranial pressure, transpulmonary thermodilution

## Abstract

Background: Prevention and treatment of haemodynamic instability and increased intracranial pressure (ICP) in patients with subarachnoid haemorrhage (SAH) is vital. This study aimed to evaluate the effects of protocolised cerebral perfusion pressure (CPP)-guided treatment on morbidity and functional outcome in patients admitted to the intensive care unit (ICU) with SAH. Methods: We performed a retrospective study comparing 37 patients who received standard haemodynamic treatment (control group) with 17 individuals (CPP-guided group) who were on the CPP-guided treatment aimed at maintaining CPP > 70 mmHg using both optimisations of ICP and mean arterial pressure (MAP). Results: MAP, cumulative crystalloid doses and fluid balance were similar in both groups. However, the incidence of delayed cerebral ischaemia was significantly lower in the CPP-guided group (14% vs. 64%, *p* < 0.01), and functional outcome as assessed by the Glasgow Outcome Scale at 30 days after SAH was improved (29.0% vs. 5.5%, *p* = 0.03). Conclusions: This preliminary analysis showed that implementing a CPP-guided treatment approach aimed at maintaining a CPP > 70 mmHg may reduce the occurrence of delayed cerebral ischaemia and improve functional outcomes in patients with SAH. This observation merits further prospective investigation of the use of CPP-guided treatment in patients with SAH.

## 1. Introduction

Subarachnoid haemorrhage (SAH) accounts for 5% of strokes and is mainly caused by ruptured intracranial aneurysms [1]. Severe SAH is associated with a severely (grade IV and V on the World Federation of Neurological Surgeons (WFNS) scale) decreased level of consciousness. These patients often present with haemodynamic instability. Following blood extravasation into the subarachnoid space, intracranial pressure (ICP) rises, restricting cerebral blood flow. Decreased cerebral blood flow triggers the cytokine storm that leads to multi-organ failure, as seen in sepsis. In addition, central dysregulation of water and electrolyte homeostasis impairs antidiuretic hormone secretion and manifests as diabetes insipidus and cerebral salt wasting syndrome. Haemodynamic instability is increased [2,3,4].

Intracranial hypertension in patients with aneurysmal subarachnoid haemorrhage (aSAH) has many causes, including cerebral oedema, hydrocephalus, intracranial haemorrhage or stroke. It occurs in approximately 70% of patients [5,6]. There is no clear consensus on ICP monitoring and treating elevated intracranial pressure in such patients, as opposed to patients with traumatic brain injury (TBI). The Brain Trauma Foundation recommends monitoring ICP and CPP (cerebral perfusion pressure) in all patients with severe TBI and implementing therapeutic interventions if ICP exceeds 22 mmHg. It is recommended to maintain CPP at 60 to 70 mmHg [7].

The mechanisms responsible for the volemic status in aSAH patients are complex, so both hypovolemia and hypervolemia can be detrimental in patients with aSAH. Treatment based on additional haemodynamic parameters, such as MAP (mean arterial pressure), CVP (central venous pressure), diuresis and fluid balance, may be insufficient. The transpulmonary thermodilution (TPTD) technique provides additional information on haemodynamic and volemic status [8].

Several international guidelines [9,10,11] advise the maintenance of normovolemia. However, these guidelines and the results of clinical trials are inconclusive as to which haemodynamic parameters may be useful in guiding therapy.

Cerebral blood flow can be adjusted using CPP measurement. Cerebral blood flow and pressure optimisation may prevent secondary brain injury, reduce mortality and improve functional outcomes in patients with SAH. This retrospective study aimed to evaluate the effects of CPP-guided management compared with conventional management on intensive care unit (ICU) mortality and functional outcome in patients with SAH.

## 2. Materials and Methods

### 2.1. Patient and Inclusion Criteria

The study was originally designed as a prospective, randomised trial of adult patients with SAH caused by ruptured intracranial aneurysms treated in the ICU. There was no validated CPP-guided protocol for managing these patients, so we designed the trial as a proof-of-concept pilot study. The study protocol was approved by the Bioethics Committee of the Medical University of Poznan (number: 450/19).

Unfortunately, due to the COVID-19 pandemic, the design of the primary study could not be realised without serious interference with the original protocols. Several mandatory changes to our ICU profile were introduced, forcing us to discontinue the project. Therefore, we decided to retrospectively analyse the available data of patients with SAH admitted to our unit between March 2019 and December 2021. For this analysis, we selected patients either treated with standard medical therapy based on clinical recommendations or whose therapy was guided by CPP and aimed at maintaining a cerebral perfusion pressure > 70 mmHg.

### 2.2. Exclusion Criteria

We used the following exclusion criteria: cardiac arrhythmias (severe bradycardia < 45 beats/min, tachycardia > 120 beats/min, atrial fibrillation or flutter, ventricular and supraventricular tachycardia, 2nd degree or complete atrioventricular conduction abnormalities), cardiac pacing, spontaneous breathing, inadequate haemodynamic measurement by TPTD, known neurological deficits before admission and short ICU stay of less than 72 h.

### 2.3. Measurements

On admission to the ICU, all patients were assessed using the SOFA (Sequential Organ Failure Assessment), APACHE II (Acute Physiology and Chronic Health Evaluation II), WFNS (World Federation of Neurological Surgeons), GCS (Glasgow Coma Scale) and Hunt–Hess scales. The extent of intracranial haemorrhage on CT (computed tomography) was assessed using the Fisher scale.

### 2.4. Study Flow and Treatment Protocol

Invasive blood pressure and central venous pressure were monitored in all patients. Dexmedetomidine infusion at a rate of 0.1–0.2 µg/kg/h and titrated doses of propofol (Fresenius Kabi, Bad Homburg, Germany) were used for sedation, while sufentanyl (Chiesi, Wien, Austria) was used for analgesia. DCI (delayed cerebral ischemia) prophylaxis was achieved by intravenous nimodipine (Bayer, Leverkusen, Germany) infusion. Ruptured aneurysm obliteration, mechanical ventilation, stress ulcer prophylaxis, antiseizure therapy, thrombo-prophylaxis, feeding, glycaemic and temperature control were based on European Stroke Organisation (ESO) guidelines [9].

In the CPP-guided group, ICP was measured using intraparenchymal sensors (Pressio 2, Sophysa) and minimally invasive cardiac output monitoring calibrated by transpulmonary thermodilution (PICCO, Getinge, Feldkirchen, Germany). In the CPP-guided group, fluid therapy, vasoactive and inotropic drugs, diuretics and osmotherapy were administered according to the treatment protocol.

**Figure 1:**
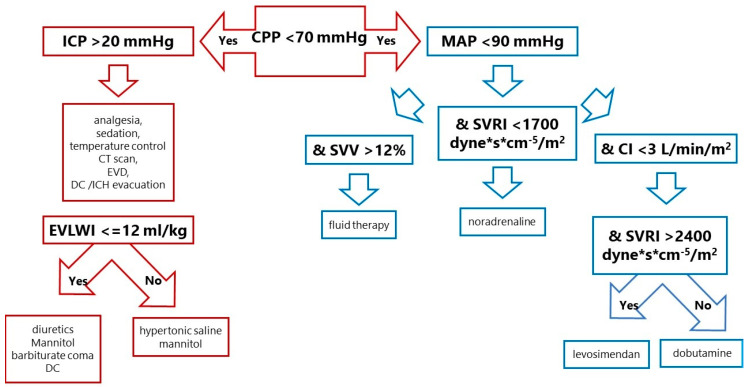
Treatment protocol in the CPP-guided group. SVV was chosen as the best parameter to assess fluid response. If the cardiac index was too low despite adequate vascular filling, dobutamine (an inotropic agent) was added. If the systemic vascular resistance index was low, vasoconstrictors (norepinephrine, dopamine, adrenaline) were chosen. When vascular resistance was too high, and there was low cardiac output, symptoms of left ventricular dysfunction such as systolic (low stroke index) and diastolic (elevated pulmonary capillary wedge pressure), then levosimendan was recommended. In SAH patients, neurogenic stunned myocardium or Takotsubo syndrome with poor left ventricular function is not rare. CPP—cerebral perfusion pressure, ICP—intracranial pressure, MAP—mean arterial pressure, EVD—extraventricular drainage, DC—decompressive craniectomy, ICH—intracranial haemorrhage, EVLWI—extravascular lung water index, SVV—stroke volume variation, SVRI—systemic vascular resistance index, CI—cardiac index.

The protocol was designed to maintain CPP above 70 mmHg. The protocol had two arms (Figure 1). The first aimed to achieve ICP values below 20 mmHg, and the second aimed to control MAP using haemodynamic parameters from cardiac output monitoring calibrated by TPTD. In the control group, only CVP, MAP, diuresis and fluid balance were used to optimise the patient’s haemodynamic status.

### 2.5. Outcomes

The primary endpoints were ICU mortality and functional outcome as assessed by the Rankin and Glasgow Outcome Scale (GOS) at 30 days.

Delayed cerebral ischaemia (DCI) is defined as neurological deterioration (a drop of at least 2 points on the GCS scale or new focal neurological deficits like haemiparesis, haemianopia or aphasia) or brain ischaemia/infarction on CT or MRI (magnetic resonance imaging) scans as a secondary outcome.

### 2.6. Statistics

The distribution of continuous data was analysed using graphical methods (histograms and Q-Q plots) and the d’Agostino–Pearson test. Because of the non-Gaussian distribution, the data were summarised using the median and the 25th (Q1) and 75th (Q3) percentiles. Comparisons between CPP-guided groups were made using the Mann–Whitney U test. The exact Fisher test was used for categorical comparisons. A *p*-value < 0.05 was considered statistically significant. Statistical analysis was performed using MedCalc (version 20.115) (MedCalc Software Ltd., Ostend, Belgium) and Graphpad Prism (Graphpad Software, La Jolla, CA, USA).

## 3. Results

A total of 64 patients with SAH were included in the retrospective analysis. Ten patients were excluded for the following reasons:

− ICP monitoring without haemodynamic monitoring (7 patients);
− TPTD monitoring without ICP measurements (3 patients).

The final analysis included 17 patients treated with CPP-guided management according to the primary design of the study and 37 patients treated with standard therapy. All ruptured intracranial aneurysms were obliterated by endovascular embolisation during the first 24 h after the onset of SAH. Almost all (93%) aneurysms were located in the anterior cerebral circulation.

There were no significant differences in clinical characteristics between the groups (Table 1).

The median admission SOFA score was 9 points, and the median APACHE II score was 18. Patients presented with worse consciousness with a median GCS of 7 points, with no statistical difference between the two groups. Most patients in both groups had severe clinical forms of SAH (Table 2) according to WFNS grade IV or V, and 95.5% were classified as Hunt–Hess grade III, IV or V (Table 2).

During the first three days after admission to the ICU, heart rate, MAP and CVP were similar in the two groups analysed (Table 3).

Values of advanced haemodynamic monitoring by TPTD in the CPP-guided group during the first 72 h were within the physiological range (Table 4). All patients, except one in the CPP-guided group (whose ICP reached 70 mmHg despite maximal treatment), had ICP values < 22 mmHg with CPP above 65 mmHg (Table 4).

### 3.1. Haemodynamic Therapy

Cumulative daily fluid intake, crystalloid infusion volume, diuresis, fluid balance and nimodipine/furosemide dosage did not differ significantly between the two groups during the first 3 days (Table 5).

Patients in the CPP-guided therapy group received more colloids on day 1 (*p* = 0.03), hypertonic saline on days 1 and 2 (*p* = 0.05) and higher doses of catecholamines (noradrenaline and dobutamine) on days 2 and 3 (*p* = 0.05).

### 3.2. Neurosurgical Treatment

Extraventricular drainage was performed in half of the patients in the CPP-guided therapy group (9 patients) and one-third of the patients in the control group (13 patients) (*p* = 0.23). Decompressive craniectomy was required in one-third of patients in the CPP-guided therapy group (4 patients) and one-sixth in the control group (5 patients) (*p* = 0.43). Overall, neurosurgical interventions were more frequent in the CPP-guided therapy group (13 patients, 76% of patients) than in the control group (17 patients, 46% of patients) (*p* = 0.04).

### 3.3. Mortality Rate, DCI and Functional Outcomes

The mortality rate reached 23% in the CPP-guided therapy group and 43% in the control group (*p* = 0.23). The incidence of DCI was significantly lower in the CPP-guided therapy group (14% vs. 64%; *p* < 0.01).

The good functional outcome assessed in the Rankin scale (0 to 3) was observed in 24% of patients in the CPP-guided therapy group and 5.5% in the control group (*p* = 0.07). In the Glasgow Outcome Scale, favourable functional outcome was noticed in 29% of patients from the CPP-guided therapy group and 5.5% in the control group (*p* = 0.03).

## 4. Discussion

This retrospective analysis shows that patients treated according to the CPP measurements had a lower incidence of DCI and better functional outcomes than those treated with standard care.

### 4.1. Epidemiology, Prognostic Scales, Mortality and Functional Outcome

Approximately 10% of SAH patients die before medical help arrives. Within the first 3 months, one-third of patients do not survive, and of those who do, one-third experience severe disability [1]. A meta-analysis of 2700 cases of severe SAH showed that 30% of patients had a favourable functional outcome 3 months after the initial bleeding episode [12]. In an observational study of 324 SAH patients, lower GCS, WFNS grade V and higher Fisher scores were associated with worse outcomes in severe SAH cases [13].

Our study showed relatively low rates of favourable functional outcomes, ranging from 6% in the control group to 30% in the CPP-guided group. Furthermore, almost all our patients presented with clinically advanced SAH, characterised by high Fisher scores, WFNS grade and low GCS. These factors are known to increase the risk of a poor functional outcome. Consequently, all these patients required ICU admission and underwent invasive haemodynamic monitoring of arterial and central venous pressures. In addition, the functional outcome of our patients was assessed on the 30th day after SAH, with some still in the ICU. Notably, SAH patients in other studies typically present with mild, moderate and severe clinical manifestations. Additionally, their clinical outcomes are usually assessed 6 months to 1 year after SAH, during which many undergo extensive rehabilitation.

Current ESO [9] and ASA/AHA [10] guidelines recommend intravascular embolisation or surgical clipping of ruptured intracranial aneurysms to prevent rebleeding as early as possible within the first 72 h. Patients with low-grade SAH benefit from early (within 24 h) or very early (within 12 h) obliteration of the aneurysm [14,15]. Our patients with ruptured intracranial aneurysms were treated endovascularly within 24 h of initial haemorrhage and 6 h of admission to the ICU.

### 4.2. Intracranial Hypertension

High levels of ICP are associated with increased mortality and poor functional outcome in patients with various central nervous system pathologies, including TBI, acute ischaemic stroke and SAH [5,6]. In patients with TBI, monitoring of ICP is recommended when GCS is <8 and cranial CT scan is abnormal. ICP monitoring is also recommended in patients with a normal CT scan and age > 40 years, systolic blood pressure < 90 mmHg or abnormal motor posture [7]. Guidelines for ICP monitoring in SAH [9,10,11] are less consistent. Few studies of intracranial hypertension in aSAH have shown that elevated ICP is a common finding, even in patients with good-grade SAH. However, elevated ICP values after ruptured aneurysm repair have been associated with a worse outcome, especially when ICP did not decrease [16]. In our study, abnormal ICP monitoring led to more ventriculostomies, decompressive craniectomies and intravenous infusions of hypertonic saline. Although it did not reduce mortality, delayed cerebral ischaemia occurred in fewer patients with better functional outcomes as assessed by the GOS scale.

### 4.3. Haemodynamic Profile

The haemodynamic profile in patients with aSAH was studied by Mutoh et al. [17]. Using TPTD, they found a hyperdynamic circulation on admission with higher CI values and lower GEDVI and ITBVI. Such conditions were associated with higher blood concentrations of epinephrine, norepinephrine and cortisol. These findings suggest sympathetic hyperreactivity in the early phase after aneurysm rupture. Sympathetic overdrive can cause systemic and pulmonary vasoconstriction. The increase in hydrostatic pressure in the pulmonary capillaries may cause fluid exudation from the intravascular space into the alveoli, manifesting as pulmonary oedema. Increased circulating catecholamines may lead to regional cardiac wall motion abnormalities, resulting in neurogenic stunned myocardium or Takotsubo syndrome [3,4].

In contrast to other studies, the haemodynamic measurements by TPTD in our patients were within the normal range during the first 3 days. Our patients were generally haemodynamically resuscitated on admission, and the ruptured aneurysms were promptly secured. In addition, our patients received small doses of dexmedetomidine to blunt the sympathetic response.

### 4.4. Haemodynamic Support

Delayed cerebral ischaemia (DCI) is a significant complication in approximately one-third of patients with aneurysmal SAH, typically between 4 and 13 days after SAH [18]. Although the pathomechanism of DCI remains unclear, it has been attributed to vasospasm resulting from extravasated blood. However, vasospasm alone does not explain all cases, as neurological deficits do not always correlate with constricted arteries, and treatments targeting vasospasm do not consistently improve outcomes. Other hypotheses include early brain injury, inflammation, microthrombosis and cortical spreading depression as potential contributors to DCI [19,20,21,22].

DCI treatment options are limited because hypervolemia and haemodilution are detrimental [23,24]. The induction of hypertension, recommended by the ASA/AHA when neurological status deteriorates, can be complicated by arrhythmias and pulmonary oedema.

In our study, the CPP-guided group received minimally invasive haemodynamic monitoring using TPTD to maintain MAP at levels sufficient to maintain a CPP of 70 mmHg. We hypothesised that this approach could improve patient survival and reduce DCI.

Previous studies have targeted hyperdynamic therapy (induced hypertension and hypervolemia) at the onset of DCI in SAH patients. Advanced haemodynamic monitoring using pulmonary artery catheters, cardiac output monitoring, pulmonary capillary wedge pressure, CVP and global end-diastolic volume index (GEDVI) were used. The intention was to guide fluid therapy and cardiac index to adjust catecholamine infusion [25,26]. While these trials did not show a significant difference in the incidence of DCI, there was a trend towards improved functional outcomes and fewer clinical cardiac complications in the targeted therapy group compared with the standard therapy group. Subgroup analysis suggested that treatment with advanced haemodynamic monitoring may reduce the incidence of DCI and the length of hospital stay. It may also improve functional outcomes in patients with low-grade SAH.

Targeted therapy was initiated on admission and continued for the first 3 days in CPP-guided SAH patients. We avoided potentially harmful hypervolemia from day one and optimised MAP to maintain a CPP of 70 mmHg. If clinically warranted, transient hypertension was induced only in cases of elevated ICP. In a recent randomised controlled trial [26], MAP was above 70 mmHg, and GEDVI was used to guide fluid therapy. Dobutamine and norepinephrine were titrated in patients with fluid refractoriness to increase cardiac index. If DCI occurred, hypertension was induced to achieve MAP > 100 mmHg. This study observed a reduced incidence of DCI and improved functional outcomes as assessed by the Glasgow Outcome Scale at three months, similar to our findings, although CPP levels were not included.

Oral nimodipine is the only effective preventive measure for DCI [23]. A randomised controlled trial by Kronvall et al. [27] showed equivalent efficacy of intravenous nimodipine. To improve the bioavailability of intravenous nimodipine, we administered it in titrated doses as long as norepinephrine doses did not exceed 0.4 mcg/kg/min.

Hyperdynamic therapy for the prevention and treatment of DCI requires intensive fluid therapy. It may be associated with a positive fluid balance and increased mortality in critically ill patients [28]. Vergouwen et al. [29] compared standard fluid therapy with a CPP-guided group receiving fluid therapy based on TPTD parameters. The control group received a mean daily fluid volume of 6 ± 1 L, while the CPP-guided group received half. Cumulative fluid doses during the first days of treatment were correlated with the incidence of DCI. Similarly, Sakr et al. [30] found that a less negative fluid balance was associated with a poor prognosis in SAH patients.

In our study, the median volume of fluid administered daily during the first three days ranged from 2500 to 2850 mL. However, the net fluid balance was negative, and there were no differences between the two groups. The CPP-guided group received more colloids, hypertonic saline and higher doses of catecholamines, but this did not result in significant differences in MAP between the two groups.

### 4.5. Limitations of the Study

Several limitations of this study must be acknowledged. First, it is a retrospective analysis with all the known limitations of such an approach. However, thousands of clinical trials begin with retrospective analyses. Although our study was originally prospective, it had to be stopped early due to the COVID-19 pandemic, compromising project design and data quality. Another limitation is using the CPP-guided protocol only for the first 72 h. This decision was influenced mainly by the need to transfer patients back to their referring institutions after endovascular embolisation and neurosurgical treatment, and in a few cases, by limited project resources and the original intention of the study as a proof-of-concept trial. Nevertheless, we cannot conclude anything about the long-term effects of CPP monitoring on patient outcomes.

In addition, haemodynamic and ICP data and pharmacological interventions are presented as daily median values. Providing more granular data would unlikely add significant new knowledge while potentially obscuring the main findings.

Finally, the relatively short follow-up period of 30 days limits the assessment of long-term neurological and clinical outcomes. Complex neurological recovery and rehabilitation may continue beyond this timeframe, limiting the ability of the study to capture the full picture of patient outcomes.

To summarise, these limitations must be considered when interpreting and extrapolating the results to clinical practice.

### 4.6. Novelty the Study

This study represents the pioneering investigation into a CPP-guided protocol’s impact on SAH patients’ outcomes. Our findings demonstrate that implementing CPP as a personalised approach to SAH treatment within the initial 72 h may reduce the occurrence of delayed cerebral ischaemia. By tailoring treatment based on individual CPP targets, optimising cerebral perfusion and mitigating the risk of secondary brain injury might be improved.

We provide initial evidence suggesting that such an approach may be clinically beneficial if applied as early as the first 72 h. Despite several limitations, our study represents a crucial first step in exploring the potential benefits of a CPP-guided approach for SAH patients.

## 5. Conclusions

Overall, the combination of ICP and haemodynamic monitoring in the CPP-guided group produced positive results in SAH patients, reducing the incidence of DCI and improving functional outcomes. These findings highlight the potential importance of implementing a comprehensive and personalised treatment strategy for patients with SAH.

Further research is warranted to confirm our findings and to elucidate the precise mechanisms by which CPP-guided therapy exerts its effects. Future studies should include larger sample sizes, longer duration of CPP monitoring and follow-up of patients after hospital discharge.

## Figures and Tables

**Table 1:** Comparison of baseline clinical characteristics of SAH patients admitted to the ICU and treated with either standard therapy (control group) or CPP-guided therapy.

	Control Group n = 37	CPP-Guided Group n = 17	
Variable	Median (Q1–Q3)	Median (Q1–Q3)	*p* Value
Age (years)	63 (57−68)	54 (48−72)	0.48
Female—number (percentage)	23 (62%)	11 (64%)	1.00
Body weight (kg)	75 970−85)	78 (65−85)	0.92
Height (cm)	165 (164−175)	168 (165−182)	0.21
BMI (kg/m^2^)	26.5 (23.9–28.4)	24.8 (23.9–28.3)	0.35
Troponin T (ng/mL)	39 (24–163)	61 (8–152)	0.68
NTproBNP (pg/mL)	1045 (251–2077)	314 (165–995)	0.08
Lactate (mmol/L)	1.2 (0.8–1.8)	1.2 (0.9–1.7)	0.96
Albumin (mg/dL)	4.1 (3.8–4.5)	4.3 (3.8–4.5)	0.5
CRP (mg/L)	4.4 (1.6–15)	3.1 (2.4–5)	0.75
Hgb (g/dL)	13.1 (11.9–13.8)	12.4 (12.6–14)	0.41
Na+ (mEq/L)	143 (140–146)	142 (138–146)	0.69
Mg++ (mg/dL)	1.9 (1.8–2.1)	2.1 (1.8–2.1)	0.46
SOFA (points)	9 (7–12)	8 (7–10)	0.32
APACHE II (points)	19 (16–25)	18 (14–21)	0.24
GCS (points)	7 (6–9)	6 (5–8)	0.52
ICH score (points)	2 (0–3)	2 (1–3)	0.15

BMI—body mass index, CRP—C-reactive protein, SOFA—Sequential Organ Failure Assessment, APACHE II—Acute Physiology and Chronic Health Evaluation II, GCS—Glasgow Coma Scale, ICH—intracranial haemorrhage.

**Table 2:** Comparisons of the rates of patients with more severe clinical forms of SAH according to the Hunt–Hess class (Hunt–Hess class III, IV or V), World Federation of Neurological Surgeons (WFNS class IV or V) and the extent of intracranial haemorrhage on computed tomography (Fisher class IV) scales between the control and CPP-guided therapy groups.

Scale	Control Group n = 37	CPP-Guided Group n = 17	*p* Value
Hunt–Hess III–V	35 (94.6%)	17 (100%)	1.0
WFNS IV–V	34 (91.9%)	16 (94.1%)	1.0
Fisher IV	30 (81.1%)	16 (94.1%)	0.4112

WFNS—World Federation of Neurological Surgeons.

**Table 3:** Comparisons of standard haemodynamic measures (MAP, HR and CVP) between patients from the control and CPP-guided therapy groups.

	Day 1			Day 2			Day 3		
	Control Group n = 37	CPP-Guided Group n = 17		Control Group n = 37	CPP-Guided Group n = 17		Control Group n = 37	CPP-Guided Group n = 17	
	Median (Q1–Q3)	Median (Q1–Q3)	*p* Value	Median (Q1–Q3)	Median (Q1–Q3)	*p* Value	Median (Q1–Q3)	Median (Q1–Q3)	*p* Value
MAP (mmHg)	88 (84–97)	90 (84–97)	0.64	90 (85–97)	93 (86–96)	0.59	93 (87–99)	93 (88–99)	0.81
HR (beats per minute)	76 (60—82)	68 (57–88)	0.63	73 (63–87)	72 (56–87)	0.46	74 (66–86)	76 (62–85)	0.7
CVP (mmHg)	8 (5–10)	9 (6–10)	0.45	8 (7–10)	8 (7–11)	0.27	8 (7–11)	8 (7–10)	0.98

MAP—mean arterial pressure, HR—heart rate, CVP—central venous pressure.

**Table 4:** Haemodynamic parameters from transpulmonary thermodilution and ICP and CPP values in patients with the CPP-guided therapy.

	Day 1	Day 2	Day 3
	Median (Q1–Q3)	Median (Q1–Q3)	Median (Q1–Q3)
CI (L/min/m^2^)	2.9 (2.6–3.2)	2.8 (2.4–3.3)	2.8 (2.5–3.6)
SVV (%)	11 (9–16)	10 (6–14)	9 (6–14)
SVRI (dyne*s*cm^−5^/m^2^)	2145 (1925–2572)	2212 (1799–2371)	2350 (1820–2660)
GEDVI (mL/m^2^)	663 (575–808)	730 (657–807)	723 (623–886)
ITBVI (mL/m^2^)	790 (767–995)	880 (801–1019)	890 (768–1120)
EVLWI (mL/kg)	8 (7–9)	9 (7–10)	9 (7–10)
PVPI	1.5 (1.4–1.7)	1.5 (1.4–1.6)	1.6 (1.4–1.8)
ICP (mmHg)	18 (10–20)	15 (10–20)	12 (8–15)
CPP (mmHg)	74 (71–80)	78 (67–80)	81 (70–88)

CI—cardiac index, SVV—stroke volume variation, SVRI—systemic vascular resistance index, GEDVI—global end-diastolic volume index, ITBVI—intrathoracic blood volume index, PVPI—pulmonary vascular permeability index, ICP—intracranial pressure, CPP—cerebral perfusion pressure.

**Table 5:** Fluid balance, diuresis, fluid therapy, osmotherapy and catecholamines.

	Control Group n = 37	CPP-Guided Group n = 17	
	Median (Q1–Q3)	Median (Q1–Q3)	*p* Value
Day 1			
Crystalloids (mL/24 h)	1500 (1000–2000)	1500 (1000–2000)	0.8469
Fluids (mL/24 h)	2480 (2110–3210)	2230 (1920–3130)	0.2759
Diuresis (mL/24 h)	2230 (1640–2940)	2080 (1600–3430)	0.6957
Fluid balance (mL/24 h)	−160 (−950–350)	−690 (−1370–150)	0.1267
Furosemide (mg/kg/24 h)	0.29 (0.02–0.53)	0.27 (0.13–0.59)	0.7360
Mannitol (g/kg/24 h)	0.43 (0–0.6)	0.46 (0.23–0.58)	0.7356
Day 2			
Crystalloids (mL/24 h)	1000 (500–1000)	1000 (1000–1000)	0.6482
Fluids (mL/24 h)	2780 (2260–3310)	2650 (2320–3120)	0.6482
Diuresis (mL/24 h)	2740 (2020–3290)	2620 (2150–3650)	0.8231
Fluid balance (mL/24 h)	−920 (−20––1520)	−870 (−1380––600)	0.5266
Furosemide (mg/kg/24 h)	0.37 (0.02–0.69)	0.56 (0.37–0.69)	0.5687
Mannitol (g/kg/24 h)	0.5 (0.17–0.6)	0.53 (0.01–0.67)	0.6461
Day 3			
Crystalloids (mL/24 h)	500 (500–1000)	500 (500–1000)	0.8724
Fluids (mL/24 h)	2855 (2250–3170)	3080 (2360–3790)	0.0993
Diuresis (mL/24 h)	3020 (2500–3440)	3000 (2460–3780)	0.7657
Fluid balance (mL/24 h)	−810 (−1390–400)	−830 (−1400–590)	0.7943
Furosemide (mg/kg/24 h)	0.51 (0.27–0.69)	0.55 (0.38–0.69)	0.8961
Mannitol (g/kg/24 h)	0.5 (0–0.6)	0.47 (0.02–0.58)	0.9925

## Data Availability

The datasets generated and/or analysed for this study are currently not publicly available due to their in other analyses. Selected data, however, are available from the corresponding author upon request.
